# Os Ácidos Graxos Poli-insaturados Ômega-6 e Ômega-3 Presentes nas Hemácias Exercem uma Influência Distinta sobre o Tamanho das Partículas de LDL e suas Alterações Estruturais

**DOI:** 10.36660/abc.20230078

**Published:** 2023-11-13

**Authors:** Gustavo Henrique Ferreira Gonçalinho, Geni Rodrigues Sampaio, Rosana Aparecida Manólio Soares-Freitas, Nágila Raquel Teixeira Damasceno

**Affiliations:** 1 Departamento de Nutrição Faculdade de Saúde Pública Universidade de São Paulo São Paulo SP Brasil Departamento de Nutrição – Faculdade de Saúde Pública – Universidade de São Paulo , São Paulo , SP – Brasil

**Keywords:** Ácidos Graxos Insaturados, Receptores de LDL Oxidado, Lipoproteínas LDL

## Abstract

**Fundamento:**

Embora os ácidos graxos poli-insaturados ômega-3 e ômega-6 (AGPIs n-3 e n-6) tenham efeitos bem conhecidos sobre os fatores de risco de doenças cardiovasculares (DCV), ainda existe um conhecimento limitado sobre como eles afetam os indicadores de qualidade da LDL.

**Objetivo:**

Avaliar as associações dos AGPIs n-3 e n-6 de hemácias com o tamanho da partícula da LDL, LDL-c pequena e densa (sdLDL-c) e com LDL eletronegativa [LDL(-)] em adultos com fatores de risco para DCV.

**Métodos:**

Estudo transversal com 335 homens e mulheres de 30 a 74 anos com, pelo menos, um fator de risco cardiovascular. Foram realizadas análises de parâmetros bioquímicos, como glicose, insulina, HbA1c, proteína C reativa (PCR), perfil lipídico, subfrações de lipoproteínas, partícula eletronegativa de LDL [LDL(-)] e seu autoanticorpo, e os AGPIs n-3 e n- 6 de hemácias. Os testes t independente/teste de Mann-Whitney, ANOVA unidirecional/teste de Kruskal-Wallis e regressões lineares múltiplas foram aplicados. Todos os testes foram bilaterais e um valor de p inferior a 0,05 foi considerado estatisticamente significativo.

**Resultados:**

A relação n-6/n-3 de hemácias foi associada ao aumento dos níveis de LDL(-) (β = 4,064; IC de 95% = 1,381 – 6,748) e sdLDL-c (β = 1,905; IC de 95% = 0,863 – 2,947), e redução do tamanho das partículas de LDL (β = -1,032; IC de 95% = -1,585 − -0,478). Individualmente, os AGPIs n-6 e n-3 apresentaram associações opostas com esses parâmetros, realçando os efeitos protetores do n-3 e evidenciando os possíveis efeitos adversos do n-6 na qualidade das partículas de LDL.

**Conclusão:**

O AGPI n-6, presente nas hemácias, foi associado ao aumento do risco cardiometabólico e à aterogenicidade das partículas de LDL, enquanto o AGPI n-3 foi associado a melhores parâmetros cardiometabólicos e à qualidade das partículas de LDL.

**Figura Central:**
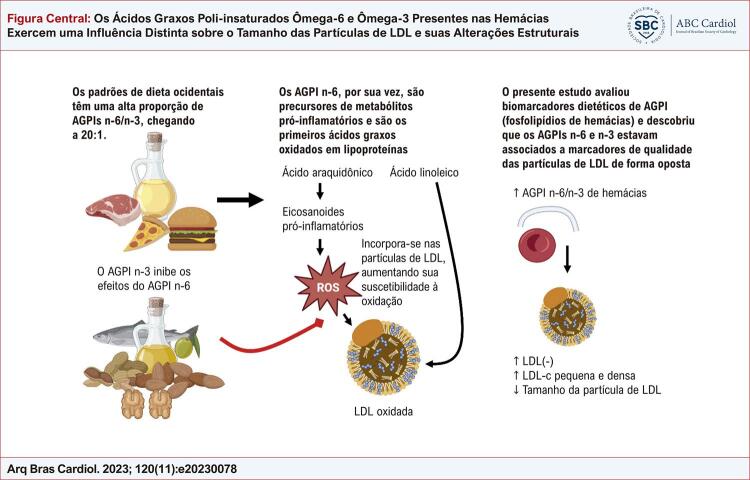
: Os Ácidos Graxos Poli-insaturados Ômega-6 e Ômega-3 Presentes nas Hemácias Exercem uma Influência Distinta sobre o Tamanho das Partículas de LDL e suas Alterações Estruturais

## Introdução

As terapias redutoras de colesterol representam a primeira linha de prevenção de complicações ateroscleróticas devido à forte relação do LDL-c com DCVs. ^1^ Apesar da LDL-c ser um excelente preditor de DCV, esta medida não reflete a qualidade das partículas de LDL. ^1^ Biomarcadores que refletem a qualidade das partículas de LDL, como LDL-c pequena e densa (sdLDL-c), LDL eletronegativa [LDL(-)], e o tamanho da partícula de LDL, são subfrações mais suscetíveis à oxidação e à progressão da aterosclerose e apresentam melhor desempenho na predição do risco de DCVs quando comparado à sua LDL-c equivalente tradicional. ^1 , 2^

Os ácidos graxos poli-insaturados ômega-3 (AGPI n-3) demonstraram efeitos benéficos sobre os parâmetros de saúde cardiometabólicos, ^3^ e outras evidências apontam para a influência do AGPI n-3 na composição lipídica das lipoproteínas e níveis de subclasses. ^4^ Os efeitos do AGPI n-3 dependerão, no entanto, do status do AGPI n-6 devido ao alto teor da família n-6 nas dietas ocidentais. ^3^ O ácido linoleico (LA; C18:2 n-6) é o AGPI n-6 mais comum na dieta, sendo convertido em ácido araquidônico (AA; C20:4 n-6), uma importante variedade precursora de metabólitos pró-inflamatórios envolvidos na fisiopatologia das DCVs. ^3 , 5^ Entretanto, vale ressaltar que nem todos os subprodutos do ácido araquidônico (AA) desencadeiam uma resposta pró-inflamatória, ^6^ e evidências indicam que os AGPIs n-6 circulantes e presentes na dieta estão correlacionados com um menor risco de ocorrência de doença arterial coronariana (DAC), acidente vascular cerebral isquêmico e mortalidade relacionada a DCVs. ^7 - 10^ Por outro lado, o LA dietético incorporado em todas as lipoproteínas pode aumentar a sua suscetibilidade à oxidação, associada à gravidade da aterosclerose. ^11 - 15^

Assim, nosso estudo avaliou a associação dos AGPIs n-3 e n-6 presente nas hemácias com o tamanho da partícula de LDL, LDL-c pequena e densa (sdLDL-c) e LDL eletronegativa [LDL(-)] em adultos com fatores de risco para DCV.

## Métodos

### Desenho do estudo e participantes

Estudo transversal que utilizou os dados iniciais do estudo CARDIONUTRI (Registro Brasileiro de Ensaios Clínicos - ReBEC: RBR-2vfhfv) que incluiu 335 participantes com os seguintes critérios de elegibilidade: indivíduos de 30 a 74 anos sem histórico de doença cardiovascular. Indivíduos com doenças graves agudas ou crônicas, doenças infecciosas, gestantes e/ou lactantes foram excluídos. Todos os participantes foram selecionados no Hospital Universitário da Universidade de São Paulo (HU-USP), e todos os procedimentos seguiram as normas estabelecidas pelo Comitê de Ética em Pesquisa do HU-USP (CAAE n.º 0063.0.207.198-11).

### Avaliação clínica e nutricional

As condições clínicas atuais e prévias foram investigadas por meio de questionários. O exame físico incluiu avaliação do índice de massa corporal (IMC), circunferência da cintura e pressão arterial. A avaliação da dieta foi obtida por meio de três recordatórios de 24 horas e realizada no software *Food Processor* (ESHA Research, OR, EUA), com posterior ajuste energético.

### Medições bioquímicas

O sangue foi coletado após jejum de 12 horas; as hemácias foram separadas do plasma por centrifugação (3.000× g por 5 minutos a 4 ºC) e subsequentemente congeladas a -80 ºC. O nível plasmático de colesterol total, HDL-c, triglicerídeos (Labtest Diagnostica S.A., MG, Brasil), apoA-I, apoB (Wako Chemicals USA Inc., Richmond, VA, EUA), glicose (Labtest Diagnostica S.A., MG, Brasil), insulina (Life Technologies, Grand Island, NY, EUA) e proteína C reativa de alta sensibilidade (PCR-us) (Diagnostic System Laboratories, Inc., Webster, Texas, EUA) foram medidos por kits comerciais. O nível de LDL-c foi calculado pela equação de Friedewald. ^16^

### Análise de subfrações de lipoproteínas

O tamanho das lipoproteínas (HDL e LDL) foi analisado utilizando o LipoprintSystem ^TM^ (Quantimetrix, Redondo Beach, CA), que se baseia na separação e quantificação de subfrações de lipoproteínas por gel de poliacrilamida não desnaturante. Sob análise eletroforética, as subfrações foram somadas para determinar a área relativa de cada subunidade de lipoproteína (expressa como porcentagem de cada subfração). Em seguida, essa relação foi multiplicada pelo nível plasmático de colesterol total para determinar a concentração de colesterol em cada subfração de LDL, e pelo colesterol em HDL para quantificar a concentração de colesterol em cada subfração de HDL. Com base nos resultados, sete subfrações de LDL foram identificadas, nas quais as subfrações LDL-1 e LDL-2 foram classificadas como grandes, e LDL-3 a LDL-7 como partículas menores e mais densas (sdLDL-c). Para a HDL, dez subfrações foram identificadas, nas quais as partículas de HDL-1 a HDL-3 foram classificadas como grandes, HDL-4 a HDL-7 como intermediárias e HDL-8 a HDL-10 como pequenas. Um ponto de corte de 25,5 nm foi utilizado para determinação dos fenótipos LDL A e não-A. O tamanho médio do LDL (nm) também foi determinado. Uma relação entre partículas grandes e pequenas de HDL foi calculada a partir da porcentagem de subfrações de lipoproteínas, como segue: (HDL-1 + HDL-2) / (HDL-9 + HDL-10). A relação para partículas grandes e pequenas de LDL foi calculada da seguinte forma: (LDL-1 + LDL-2) / LDL-3 a LDL-7).

### Análise eletronegativa de LDL e autoanticorpos

A detecção de LDL eletronegativa [LDL(-)] e de autoanticorpos anti-LDL(-) foi realizada de acordo com método publicado anteriormente. ^17^ A LDL(-) eletronegativa foi detectada por meio do método ELISA sanduíche. As placas de microtitulação de poliestireno de fundo plano, com 96 poços (Costar, Corning Inc, NY, EUA), foram revestidas com 0,5 mg/poço de anticorpo monoclonal anti-LDL (-) 1A3H2 em 50 mL/poço de tampão carbonato-bicarbonato 0,05 M, pH 9,6, a 4 ºC, durante a noite. As placas foram lavadas três vezes com PBS, pH 7,4, contendo 0,05% de Tween-20 (200 mL/poço). Posteriormente, os sítios de ligação livres foram bloqueados pela adição de 150 mL/poço de PBS contendo 2% de leite em pó desnatado, previamente inativado por aquecimento (100 ºC), e 0,01% de Tween-20 por 1,5 h a 37 ºC, seguido de lavagem conforme mencionado acima. Padrão ou plasma (1:2000 diluído em PBS contendo 1% de leite desnatado e 0,01% de Tween-20) foi adicionado (50 mL/poço) nas placas e incubado por 1,5 h a 37 ºC. Em seguida, as placas foram lavadas e incubadas com 0,5 mg/poço de anticorpo monoclonal anti-LDL(-) 2C7D5F10 conjugado com biotina por uma hora a 37 ºC. Posteriormente, 50 mL/poço de peroxidase de rábano conjugada com estreptavidina (Invitrogen Corp., Carlsbad, CA, EUA) foram adicionados e incubados por uma hora a 37 ºC. A reatividade da peroxidase foi medida em placas lavadas por incubação com 50 mL de ortofenilenodiamina (OPD) diluída em tampão citrato fosfato, pH 5,3, a 37 ºC por 15 min. A reação foi interrompida pela adição de ácido sulfúrico 2 M, e a absorbância a 492 nm foi medida por espectrofotometria, usando um leitor de microplacas (Spectra Count Microplate Photometer, Packard Instruments Company, Downers Grove, IL, EUA). Uma curva de calibração (de 0,6 a 20 mg/mL) foi construída com LDL(-) humano obtido por FPLC usando uma coluna iônica. Os resultados foram expressos em U/L, com unidades representando 1 g/L de equivalente de apolipoproteína B oxidada.

### Análise de ácidos graxos de hemácias

A análise de ácidos graxos (AG) das membranas eritrocitárias foi realizada com base em método previamente descrito. ^18^ Após a separação do plasma (3.000× *g* , 10 min, 4 °C), 300 µL de eritrócitos foram lavados com 5 mL de solução salina tamponada com fosfato (PBS) (pH 7,4) quatro vezes. O precipitado foi transferido para tubos rosqueados, aos quais foram adicionados 1,75 mL de metanol, 50 µL de solução padrão interno contendo 1 mg de ácido tridecanóico (C13:0)/1 mL de hexano e 100 µL de cloreto de acetila. Em seguida, a solução foi agitada em vórtex e aquecida em banho-maria a 90 °C por 1 hora. Na sequência, adicionou-se 1,5 mL de hexano e a solução foi homogeneizada por 1 min. As amostras foram centrifugadas a 1500× *g* , 4 °C, por 2 min, e 800 µL do sobrenadante foram transferidos para um tubo diferente. Esta etapa foi repetida com a adição de 750 µL de hexano. Os tubos contendo os sobrenadantes coletados foram transferidos para um concentrador centrífugo a 40 °C, por 20 min. Em seguida, os ésteres metílicos de AG foram dissolvidos em 150 µL de hexano e transferidos para um inserto de vidro em um frasco, que posteriormente foi enviado para análise por cromatografia gasosa (Shimadzu CG-2010 equipado com coluna capilar DB-FFAP, Agilent Technologies).

Os resultados foram expressos como percentual do total de AG. As análises foram realizadas considerando os ácidos graxos individualmente, bem como o n-3 total, formado pela soma do ácido α-linolênico (ALA; C18:3 n-3), ácido eicosapentaenoico (EPA; C20:5 n-3) e ácido docosahexaenoico (DHA; C22:6 n-3), e n-6 total, formado pela soma de ácido linoleico (LA; C18:2 n-6), ácido γ-linolênico (GLA; C18:3 n-6 ), ácido eicosadienoico (EDA; C20:2 n-6), ácido di-homo-γ-linolênico (DGLA; C20:3 n-6), ácido araquidônico (AA; C20:4 n-6) e ácido di-homo-linolênico (DLA; C22:2 n-6).

### Análise estatística

O presente estudo utilizou amostragem de conveniência baseada em dados secundários de um ensaio clínico randomizado anterior (ReBEC: RBR-2vfhfv).

As variáveis contínuas foram expressas por meio de média ± desvio padrão (DP) ou mediana e intervalo interquartil (IIQ), conforme distribuição dos dados, e as variáveis categóricas foram expressas por meio de frequência absoluta (n) ou relativa (%). O teste de Kolmogorov-Smirnov foi realizado em variáveis contínuas para avaliar a distribuição. Os dados relativos das características da amostra foram obtidos por meio de teste t independente ou teste U de Mann-Whitney para variáveis contínuas de acordo com a normalidade dos dados e teste qui-quadrado para parâmetros categóricos. Os pacientes foram divididos em tercis de AG de hemácias (n-3 total, n-6 total e razão n-6/n-3) e, em seguida, foram aplicados testes ANOVA unidirecional ou Kruskal-Wallis com base na distribuição da variável, usando Bonferroni como teste posthoc.

Regressões lineares múltiplas foram realizadas para associar AGs com LDL(-), tamanho de LDL e sdLDL-c, sendo esta última a variável dependente. Todos os modelos foram ajustados por idade, sexo, tabagismo e uso de estatinas. Todas as suposições da regressão foram cumpridas (ou seja, sem multicolinearidade, homocedasticidade, erros normalmente distribuídos e independentes, independência das variáveis de resultado e linearidade das variáveis).

Todos os testes foram bilaterais, com p < 0,05 considerado significativo, e realizados no software SPSS versão 20.0.

## Resultados

As características dos participantes estão descritas na Tabela 1 e na Tabela Complementar 1. As mulheres mostraram maior frequência de tratamento com estatinas e apresentaram níveis plasmáticos de colesterol total, HDL-c, LDL-c, apoA-I, HDL-c grande, intermediário e pequeno, LDL(-) e hs-CRP em comparação com os homens. Em contraste, os homens apresentaram triglicerídeos plasmáticos, sdLDL-c e glicose mais elevados. Em comparação com os homens, as mulheres apresentaram maior ingestão de lipídios (valores absolutos, mas não ingestão relativa), DGLA e EPA e menos energia (Tabela Complementar 2). O perfil de AG de hemácias analisado está descrito na Tabela Complementar 3.

**Tabela 1:** – Características da amostra

Variáveis	Total (n = 335)	

	Média/mediana ou n	DP/IIQ ou %
Idade (anos)	52,4	10,5
Atividade física (pontos)	7,2	1,4
Pressão arterial sistólica (mmHg)	133	18,3
Pressão arterial diastólica (mmHg)	81,1	10
IMC (kg/m ^2^ )	30,9	5,7
Circunferência da cintura (cm)	100,6	13,6
**Etnia**		
Branca	226	67,5
Outras	109	12,5
**Tabagismo**		
Tabagista	64	19,1
Não tabagista	271	80,9
**Consumo de álcool**		
Sim	66	19,7
Não	269	80,3
**Escolaridade**		
Ensino médio completo ou incompleto	191	57
Ensino superior completo	144	43
**Doenças não transmissíveis**		
Diabetes melito	66	19,7
Hipertensão	189	56,4
Hipotireoidismo	43	12,8
Dislipidemia	186	55,5
**Medicamentos**		
Estatinas	95	28,4
Anti-hipertensivos	169	50,4
Anti-hiperglicêmicos	69	20,6
Fibratos	9	2,7
**Características bioquímicas**		
Colesterol total (mg/dL)	204,4	42
HDL-c (mg/dL)	36	30,0-43,0
LDL-c (mg/dL)	137,2	38,7
Triglicerídeos (mg/dL)	130	97,0-190,0
Não-HDL-c (mg/dL)	167,8	41,3
ApoA-I (mg/dL)	132,8	26
ApoB (mg/dL)	103,9	24,8
HDL-c grande (mg/dL)	10	7,0-14,0
HDL-c intermediário (mg/dL)	18	15,0-21,0
HDL-c pequeno (mg/dL)	7,6	3
sdLDL-c (mg/dL)	3	1,0-9,0
lbLDL-c (mg/dL)	52,9	16,4
Tamanho da LDL (Å)	270	266,0-272,0
LDL(-) (U/L)	5,3	1,8-17,9
Anti-LDL(-) (μg/mL)	8,1	5,00-11,5
Glicose (mg/dL)	98	91,0-108,0
Insulina (μUI/mL)	16,1	12,7-22,1
HbA1c (%)	5	4,7-5,3
Adiponectina (μg/mL)	8,3	4,7-13,0
Leptina (ng/mL)	34,6	11,0-65,4
hs-CRP (mg/L)	2,7	2,7-5,8

Os dados são apresentados em médias (desvio padrão) ou medianas (intervalo interquartil) dependendo da distribuição para dados contínuos e valor absoluto (n) e frequência (%) para dados categóricos. ApoA-I: apolipoproteína A-I; ApoB: apolipoproteína B; IMC: índice de massa corporal; hs-CRP: proteína C reativa de alta sensibilidade; lbLDL-c: colesterol LDL grande e flutuante; LDL(-): LDL eletronegativa.

Indivíduos do terceiro tercil do n-3 total apresentaram níveis plasmáticos significativamente mais baixos de lipoproteínas plasmáticas associadas a apoB, colesterol, triglicerídeos e apoB. Além disso, melhores aspectos qualitativos de LDL foram observados neste grupo, como menor sdLDL-c (p = 0,001) e maior tamanho de partícula de LDL (p = 0,003) (Tabela Complementar 4).

As associações encontradas com AGPI n-6 total de hemácias foram comparadas de forma oposta com AGPI n-3 (Tabela Complementar 5). Verificou-se que os indivíduos do terceiro tercil apresentavam níveis plasmáticos mais elevados de colesterol total, LDL-c, não-HDL-c, apoB e apoA-I. Quanto à qualidade das partículas de LDL, o terceiro tercil apresentou maiores níveis plasmáticos de sdLDL-c (p = 0,004), lbLDL-c (p = 0,037) e LDL(-) (p = 0,002). Além disso, o tamanho médio das partículas de LDL foi menor neste tercil (p = 0,026). Associações semelhantes foram observadas com o terceiro tercil da relação n-6/n-3 de hemácias (Tabela Complementar 6).

As associações dos AGPIs n-3 e n-6 com a qualidade das partículas de LDL são mostradas na Tabela 2 . O EPA foi inversamente associado ao LDL(-). O ALA foi significativamente associado à menor sdLDL-c e maior tamanho de partícula de LDL, influenciando a associação do n-3 total com esses parâmetros. Em relação aos AGPIs n-6, o n-6 total teve associações positivas com LDL(-) e sdLDL-c, embora uma associação inversa com o tamanho das partículas de LDL tenha sido observada. Entre as associações com LDL(-), DGLA e AA apresentaram associações positivas significativas, enquanto DLA apresentou associação inversa. Entre as associações de sdLDL-c, LA, GLA, DGLA e AA apresentaram associações positivas. Além disso, o tamanho das partículas de LDL foi inversamente associado apenas com LA e DGLA entre os AGPIs n-6. Por fim, a relação de AGPIs n-6/n-3 de hemácias foi positivamente associada ao LDL(-) e ao sdLDL-c e inversamente associada ao tamanho das partículas de LDL.

**Tabela 2:** – Associações entre ácidos graxos da membrana de hemácias e LDL eletronegativa, LDL pequena e densa e tamanho de partícula de LDL

Variáveis	LDL(-)				

	R ^**2**^	β	EP	Valor de p	IC de 95%
C18:2 n-6 (LA)	0,034	0,551	0,779	0,480	-0,981 ₋ 2,083
C18:3 n-6 (GLA)	0,055	-6,071	3,643	0,097	-13,238 ₋ 1,096
C20:2 n-6 (EDA)	0,050	-7,125	7,121	0,318	-21,134 ₋ 6,885
C20:3 n-6 (DGLA)	0,065	10,724	4,253	**0,012**	2,356 ₋ 19,091
C20:4 n-6 (AA)	0,117	2,999	0,588	**< 0,001**	1,842 ₋ 4,157
C22:2 n-6 (DLA)	0,068	-12,869	4,760	**0,007**	-22,233 ₋ -3,506
Total n-6	0,075	1,163	0,367	**0,002**	0,440 ₋ 1,886
C18:3 n-3 (ALA)	0,054	-1,734	1,126	0,124	-3,949 ₋ 0,480
C20:5 n-3 (EPA)	0,061	-21,517	9,711	**0,027**	-40,621 ₋ -2,413
C22:6 n-3 (DHA)	0,048	-0,523	1,146	0,649	-2,777 ₋ 1,732
Total n-3	0,055	-1,443	0,850	0,090	-3,115 ₋ 0,229
Relação n-6/n-3	0,072	4,064	1,364	**0,003**	1,381 ₋ 6,748
	**sdLDL-c**				

	**R ^**2**^**	**β**	**EP**	**Valor de p**	**IC de 95%**
C18:2 n-6 (LA)	0,053	0,948	0,301	**0,002**	0,357 ₋ 1,540
C18:3 n-6 (GLA)	0,039	3,105	1,422	**0,030**	0,307 ₋ 5,903
C20:2 n-6 (EDA)	0,025	-0,263	2,792	0,925	-5,756 ₋ 5,230
C20:3 n-6 (DGLA)	0,049	4,824	1,654	**0,004**	1,569 ₋ 8,078
C20:4 n-6 (AA)	0,037	0,491	0,237	**0,039**	0,025 ₋ 0,958
C22:2 n-6 (DLA)	0,030	-2,405	1,835	0,191	-6,015 ₋ 1,205
Total n-6	0,053	0,453	0,144	**0,002**	0,170 ₋ 0,736
C18:3 n-3 (ALA)	0,052	-1,340	0,437	**0,002**	-2,199 ₋ -0,481
C20:5 n-3 (EPA)	0,031	-5,492	3,827	0,152	-13,020 ₋ 2,036
C22:6 n-3 (DHA)	0,026	-0,283	0,447	0,527	-1,163 ₋ 0,597
Total n-3	0,049	-0,958	0,329	**0,004**	-1,606 ₋ -0,311
Relação n-6/n-3	0,062	1,905	0,530	**< 0,001**	0,863 ₋ 2,947
	**Tamanho da partícula de LDL**				

	**R ^**2**^**	**β**	**EP**	**Valor de p**	**IC de 95%**
C18:2 n-6 (LA)	0,079	-0,504	0,160	**0,002**	-0,818 ₋ -0,190
C18:3 n-6 (GLA)	0,060	-1,313	0,758	0,084	-2,805 ₋ 0,179
C20:2 n-6 (EDA)	0,051	0,069	1,485	0,963	-2,851 ₋ 2,990
C20:3 n-6 (DGLA)	0,065	-1,955	0,884	**0,028**	-3,695 ₋ -0,215
C20:4 n-6 (AA)	0,061	-0,232	0,126	0,067	-0,480 ₋ 0,017
C22:2 n-6 (DLA)	0,055	1,007	0,977	0,303	-0,914 ₋ 2,928
Total n-6	0,075	-0,224	0,077	**0,004**	-0,374 ₋ -0,073
C18:3 n-3 (ALA)	0,077	0,699	0,232	**0,003**	0,242 ₋ 1,156
C20:5 n-3 (EPA)	0,060	3,465	2,032	0,089	-0,533 ₋ 7,462
C22:6 n-3 (DHA)	0,053	0,157	0,238	0,511	-0,311 ₋ 0,624
Total n-3	0,075	0,510	0,175	**0,004**	0,165 ₋ 0,854
Relação n-6/n-3	0,089	-1,032	0,281	**< 0,001**	-1,585 ₋ -0,478

Modelos de regressão linear múltipla ajustados por idade, sexo, tabagismo e uso de estatinas. AA: ácido araquidônico; ALA: ácido α-linolênico; DGLA: ácido dihomo-γ-linolênico; DHA: ácido docosahexaenoico; DLA: ácido di-homo-linolênico; EDA: ácido eicosadienoico; EPA: ácido eicosapentaenoico; GLA: ácido γ-linolênico.

## Discussão

O presente estudo mostrou que os AGPIs n-6 e n-3 de hemácias exerceram influências opostas nos marcadores de saúde cardiometabólicos e nas características das partículas de LDL.

Nossos resultados mostraram que indivíduos com níveis de AGPI n-6 de hemácias e relação n-6/n-3 mais elevados, e níveis de AGPI n-3 mais baixos, apresentaram níveis plasmáticos mais elevados de colesterol (total, LDL-c e não-HDL-c), apoB, apoA-I e triglicerídeos. Estes resultados são consistentes com estudos que mostram que uma elevada relação n-6/n-3 foi associada ao aumento da lipogênese e dos lípidos no sangue. ^19 - 24^

Estudos clínicos de biomarcadores de AGPI n-6 e DCV na literatura mostram resultados inconclusivos. Em um estudo anterior, um padrão de AG de hemácias, contendo mais AGPI n-6, foi um preditor independente de classificação de risco cardiovascular mais elevada associada a fatores de risco estabelecidos. ^18^ Por outro lado, AGPIs n-6 teciduais ou sanguíneos não foram associados ao risco cardiovascular. ^25 , 26^ Além disso, estudos mostraram que altos níveis de AGPI n-6, especialmente LA, eram fatores de proteção contra a síndrome coronariana aguda. ^27 - 29^ Os autores de um desses estudos afirmaram que o consumo de AGPI n-6 deve ser incentivado, apesar do baixo consumo encontrado em um estudo. ^29^ Além disso, vale ressaltar que o risco de desfechos cardiovasculares mostrou uma associação em forma de U com AA, ^28^ e o LA não foi significativamente associado ao risco de infarto do miocárdio em um desses estudos, ^29^ dificultando a conclusão dos autores sobre o real efeito do AGPI n-6 nas DCVs.

As evidências sugerem que os AGPIs n-6 reduzem o nível plasmático do colesterol, particularmente quando o AGS é substituído, sendo favorável para a redução do risco de DCV, ^30 - 32^ mas os efeitos sobre os resultados permanecem inconclusivos. ^31^ Uma metanálise de ensaios clínicos randomizados (ECR) mostrou efeitos protetores do LA nas DCVs usando óleos vegetais, que também são fonte de ALA. ^33^ Curiosamente, um ECR relatou um risco aumentado de DCV e mortalidade por todas as causas após a suplementação de AGPI n-6. ^33^ Além disso, os resultados do *Lyon Diet Heart Study* indicaram que a redução do consumo de AGPI n-6 para menos de 5% da ingestão total de energia, abaixo das recomendações dos EUA de 5-10%, resultou em uma diminuição na mortalidade total e cardiovascular, sugerindo a existência de um possível risco associado ao consumo de AGPI n-6. ^34 , 35^

Em relação aos AGPIs n-3, as evidências clínicas recentes de DCV são mais consistentes. Estudos observacionais com AGPI n-3 de hemácias ^18 , 36 - 40^ e ensaios clínicos randomizados ^41 - 43^ mostraram que o AGPI n-3 reduz o risco de DCV. Nossos resultados corroboram o efeito cardioprotetor dos AGPIs n-3, mas mostram forte influência do ALA devido à baixa ingestão de EPA e DHA (Tabela Complementar 1). Os resultados do nosso estudo estão alinhados com uma revisão sistemática e metanálise dose-resposta, que mostrou que uma maior ingestão de ALA foi significativamente associada a 10%, 8% e 11% de mortalidade por todas as causas, DCV e DAC, respectivamente. ^44^

Os fatores de risco cardiovasculares tradicionais não refletem toda a complexidade da doença aterosclerótica, tanto que os fatores de risco cardiovasculares emergentes ganharam destaque na literatura, ^18 , 45 - 48^ e os efeitos dos AGPIs nas DCV estão além desses fatores. ^18^ Entre os diversos fatores de risco não tradicionais, o sdLDL-c demonstrou ser um melhor preditor de risco de DAC do que o LDL-c. ^1^ O presente estudo descobriu que os AGPIs n-6 das hemácias e a relação n-6/n-3 estavam positivamente associados ao tamanho médio menor das partículas de LDL e a níveis mais elevados de sdLDL-c. Associações semelhantes foram observadas com LA, GLA, DGLA e AA individualmente. Em contraste com nossos achados, um estudo transversal mostrou que os AGPIs n-6 circulantes estavam modestamente associados ao tamanho do LDL. ^49^

Por outro lado, os AGPIs n-3 foram associados a maiores tamanhos de partículas de LDL e a níveis mais baixos de sdLDL-c. Tais associações corroboram os resultados de ensaios anteriores, nos quais os AGPIs n-3 diminuíram o sdLDL-c e aumentaram o tamanho das partículas de LDL. ^50 - 53^ Essas associações podem estar ligadas ao TG. TGs mais elevados aumentam a apoC-III, dificultando a depuração de lipoproteínas que contêm apoE, e resultando em aumento dos níveis circulantes de sdLDL. ^1^ Além disso, os AGPIs n-3 reduzem os níveis de apoC-III em VLDL, diminuindo os níveis de sdLDL. ^54^

Observamos também que a relação n-6/n-3 de hemácias, AGPI n-6 total, DGLA e AA foram positivamente associados ao LDL (-), enquanto o EPA e o DLA apresentaram uma associação inversa. O LDL(-), intimamente relacionado ao oxLDL e ao sdLDL, possui propriedades aterogênicas, e níveis aumentados dessa partícula são encontrados em condições de estresse oxidativo e doenças não transmissíveis. ^1 , 2^ Níveis elevados de n-6/n-3 nos tecidos estão associados ao aumento da peroxidação lipídica, ^19 - 21^ e os AGPIs n-6 nas partículas de LDL, principalmente LA e AA, são mais facilmente oxidados, resultando no aumento da formação de partículas de LDL modificadas. ^12^ Outro estudo mostrou que a redução do AGPI n-6 na dieta reduz seus metabólitos gerados pela peroxidação. ^55^ Curiosamente, descobrimos que o EPA era um fator de proteção. Isto pode ser devido ao mecanismo antiinflamatório e antioxidante do EPA e seus metabólitos, que diminuem a modificação das partículas de LDL. ^56^

Entre as limitações, não podemos inferir causalidade devido à natureza transversal do estudo. Além disso, os AGPIs de hemácias do nosso estudo diferem dos resultados anteriores. ^48 , 55 , 57^ Isto pode ser devido à falta de padronização da análise de AG circulante. Os AG das hemácias são expressos em percentagens do AG total, o que significa que os níveis de AG dependem dos AG analisados pelo método. Outro fator que pode explicar esta divergência é que o estresse oxidativo diminui a quantidade de AGPI incorporados aos tecidos. ^58^ Por fim, nosso estudo não possui uma amostra representativa e, portanto, a AG de hemácias e sua associação com fatores de risco cardiovascular podem diferir de outras populações.

Nossos resultados aumentam o conhecimento disponível sobre ácidos graxos e risco cardiovascular, especialmente em relação aos AGPIs n-6. A literatura propõe que uma maior ingestão de AGPI n-6 pode beneficiar os indivíduos em relação ao risco cardiovascular. ^7 - 10^ Contudo, os estudos apresentam resultados altamente variáveis devido à avaliação de diversos biomarcadores (como plasma, hemácias, tecido adiposo e dieta) e à ampla variação na alimentação das pessoas entre as diferentes regiões estudadas, incluindo países ocidentais e asiáticos. Com a associação de AGPI n-6 de hemácias e a relação n-6/n-3 com LDL(-) demonstrada, nosso estudo corrobora com experimentos de oxidação de LDL que mostraram que os AG oxidados são da família do AGPI n-6, ^11 - 14^ sugerindo que o consumo excessivo de AGPI n-6 pode causar efeitos adversos. Isso chama a atenção para o fato de que os benefícios identificados do AL e de outros AGPIs n-6 na literatura provavelmente dependem do estado nutricional de AGPIs n-3, uma vez que mostramos associações metabólicas favoráveis com este último.

Por fim, a presente pesquisa evidencia que estudos futuros sobre o estado nutricional dos AGPIs devem considerar ambas as famílias de ácidos graxos, uma vez que a análise por si só pode ignorar fatores de confusão. Mais estudos são necessários para avaliar a associação de AGPIs de hemácias com fatores de risco e desfechos cardiovasculares, bem como fatores de risco emergentes, como subfrações de LDL.

## Conclusão

Nosso estudo mostrou que os AGPIs n-6 de hemácias estavam associados com fatores de risco cardiometabólico desfavoráveis, bem como níveis mais elevados de sdLDL-c e LDL(-), sugerindo que um alto consumo de n-6 na dieta pode aumentar a susceptibilidade da LDL a modificações oxidativas. Por outro lado, os AGPIs ômega-3 das hemácias, especialmente ALA e EPA, demonstraram associações benéficas para a saúde cardiovascular, manifestando níveis mais baixos de sdLDL-c e LDL(-) e maior tamanho da partícula de LDL.

Consequentemente, a ingestão do AGPI n-6 (LA ou AA) deve ser reduzida, ao passo que a ingestão de AGPIs n-3 deve ser estimulada, uma vez que uma relação mais elevada de n-6 para n-3 nas hemácias e uma maior concentração de AGPI n-6 foram associados a uma menor qualidade das partículas de LDL e a fatores de risco cardiometabólicos desfavoráveis.

## Material suplementar

Para tabelas suplementares, por favor, clique aqui .
