# Supplementary material for: Os Ácidos Graxos Poli-insaturados Ômega-6 e Ômega-3 Presentes nas Hemácias Exercem uma Influência Distinta sobre o Tamanho das Partículas de LDL e suas Alterações Estruturais
Source: Arq Bras Cardiol. 2023 Nov 13;120(11):e20230078. [Article in Portuguese] doi: 10.36660/abc.20230078 (PMC10697675; doi:10.36660/abc.20230078)
Supplement: Translation

# Translation: Red Blood Cells’ Omega-6 and Omega-3 Polyunsaturated Fatty Acids Have a Distinct Influence on LDL Particle Size and its Structural Modifications

**Authors:** Gustavo Henrique Ferreira Gonçalinho, Geni Rodrigues Sampaio, Rosana Aparecida Manólio Soares-Freitas, Nágila Raquel Teixeira Damasceno

**Affiliations:** 1 Brazil Departamento de Nutrição – Faculdade de Saúde Pública – Universidade de São Paulo, São Paulo, SP – Brazil

**Keywords:** Fatty Acids, Unsaturated, Oxidized LDL Receptors, Lipoproteins, LDL

## Abstract

**Background:**

While Omega-3 and omega-6 polyunsaturated fatty acids (n-3 and n-6 PUFAs) have established effects on cardiovascular disease (CVD) risk factors, little is known about their impacts on LDL quality markers.

**Objective:**

To assess the associations of n-3 and n-6 PUFA within red blood cells (RBC) with LDL particle size, small dense LDL-c (sdLDL-c), and electronegative LDL [LDL(-)] in adults with CVD risk factors.

**Methods:**

Cross-sectional study involving 335 men and women aged 30 to 74 with at least one cardiovascular risk factor. Analyses were conducted on biochemical parameters, such as glucose, insulin, HbA1c, C-reactive protein (CRP), lipid profile, lipoprotein subfractions, electronegative LDL particle [LDL(-)] and its autoantibody, and RBC n-3 and n-6 PUFAs. Independent t-test/Mann-Whitney test, one-way ANOVA/Kruskal-Wallis test, and multiple linear regressions were applied. All tests were two-sided, and a p-value of less than 0.05 was considered statistically significant.

**Results:**

The RBC n-6/n-3 ratio was associated with increased LDL(-) (β = 4.064; 95% CI = 1.381 – 6.748) and sdLDL-c (β = 1.905; 95% CI = 0.863 – 2.947) levels, and reduced LDL particle size (β = -1.032; 95% CI = -1.585 − -0.478). Separately, n-6 and n-3 PUFAs had opposing associations with those parameters, reinforcing the protective effects of n-3 and showing the potential negative effects of n-6 on LDL particle quality.

**Conclusion:**

RBC n-6 PUFA was associated with increased cardiometabolic risk and atherogenicity of LDL particles, while n-3 PUFA was associated with better cardiometabolic parameters and LDL particle quality.

**Central Illustration:** : Red Blood Cells’ Omega-6 and Omega-3 Polyunsaturated Fatty Acids Have a Distinct Influence on LDL Particle Size and its Structural Modifications

## Introduction

Cholesterol-lowering therapies are the first-line conducts for preventing atherosclerotic complications due to the strong relationship of LDL-c with CVD.^1^Despite LDL-c being an excellent predictor of CVD, this measure does not reflect the quality of LDL particles.^1^Biomarkers that reflect LDL particle quality, such as small dense LDL-c (sdLDL-c), electronegative LDL [LDL(-)], and LDL particle size, are subfractions that are more susceptible to oxidation and atherosclerosis progression and have better performance in CVD risk prediction when compared to its traditional counterpart LDL-c.^1 , 2^

The omega-3 polyunsaturated fatty acids (n-3 PUFA) have demonstrated beneficial effects on cardiometabolic health parameters,^3^and there is further evidence that n-3 PUFA influence lipoprotein lipid composition and subclasses levels.^4^The effects of n-3 PUFA will, however, depend on the n-6 PUFA status due to the high content of the n-6 family in Western diets.^3^Linoleic acid (LA; C18:2 n-6) is the most common dietary n-6 PUFA and is converted to arachidonic acid (AA; C20:4 n-6), an important precursor variety of proinflammatory metabolites involved in the pathophysiology of CVD.^3 , 5^However, not all AA byproducts exert a proinflammatory response,^6^and evidence showed that circulating and dietary n-6 PUFA were associated with lower risks of coronary artery disease (CAD), ischemic stroke, and CVD mortality.^7 - 10^Conversely, dietary LA incorporated into all lipoproteins may increase their susceptibility to oxidation, which is associated with atherosclerosis severity.^11 - 15^

Therefore, our study evaluated the association of RBC n-3 and n-6 PUFA with LDL particle size, small dense LDL-c (sdLDL-c), and electronegative LDL [LDL(-)] in adults with CVD risk factors.

## Methods

### Study design and participants

A cross-sectional study that used the baseline data from the CARDIONUTRI study (Brazilian Clinical Trial Registry - ReBEC: RBR-2vfhfv) that included 335 participants with the following eligibility criteria: individuals aged 30 to 74 without previous history of cardiovascular disease. Those with acute or chronic severe diseases, infectious diseases, pregnant and/or lactating women were excluded. All participants were selected at the University Hospital of the University of São Paulo (HU-USP), and all procedures followed the rules established by the Research Ethics Committee of HU-USP (CAAE No. 0063.0.207.198-11).

### Clinical and nutritional assessment

Current and past clinical conditions were investigated through questionnaires. Physical examination included body mass index (BMI) assessment, waist circumference, and blood pressure. Dietary intake was obtained through three 24-hour recalls and assessed in the Food Processor software (ESHA Research, OR, USA), with subsequent energy adjustment.

### Biochemical measurements

Blood was drawn after fasting for 12 hours; RBCs were separated from plasma by centrifugation (3,000× *g* for 5 minutes at 4 ºC), then frozen at -80 ºC. Plasma total cholesterol, HDL-c, triglycerides (Labtest Diagnostica S.A., MG, Brazil), apoA-I, apoB (Wako Chemicals USA Inc., Richmond, VA, USA), glucose (Labtest Diagnostica S.A., MG, Brazil), insulin (Life Technologies, Grand Island, NY, USA), and high-sensitivity C-reactive protein (hs-CRP) (Diagnostic System Laboratories, Inc., Webster, Texas, USA) were measured by commercial kits. The LDL-c level was calculated by Friedewald equation.^16^

### Lipoprotein subfractions analysis

Lipoprotein size (HDL and LDL) was analyzed using LipoprintSystem^TM^(Quantimetrix, Redondo Beach, CA), which is based on the separation and quantification of lipoprotein subfractions by non-denaturing polyacrylamide gel. In light of electrophoresis, the subfractions were integrated to determine the relative area of each lipoprotein subunit (percentage of each subfraction), which was then multiplied by the plasma total cholesterol to calculate the cholesterol concentration in each LDL subfraction, and by the cholesterol in HDL to quantify the cholesterol concentration in each HDL subfraction. Based on the results, seven LDL subfractions could be identified, in which LDL-1 and LDL-2 subfractions were classified as large, and LDL-3 to LDL-7 as smaller and denser particles (sdLDL-c). In terms of HDL, ten subfractions were identified, in which HDL-1 to HDL-3 particles were classified as large, HDL-4 to HDL-7 as intermediate, and HDL-8 to HDL-10 as small. A cut-off point of 25.5 nm was used for determining LDL A and non-A phenotypes. The mean LDL size (nm) was also determined. A ratio between large and small HDL particles was calculated from the percentage of lipoprotein subfractions as follows: (HDL-1 + HDL-2) / (HDL-9 + HDL-10). The ratio for large and small LDL particles was calculated as follows: (LDL-1 + LDL-2) / LDL-3 to LDL-7).

### Electronegative LDL and autoantibodies analysis

The electronegative LDL [LDL(-)] and the autoantibody anti-LDL(-) detection were performed according to a previously published method.^17^The electronegative LDL(-) was detected through sandwich ELISA. The 96-well flat-bottom polystyrene microtiter plates (Costar, Corning Inc, NY, USA) were coated with 0.5 mg/well of anti-LDL (–) 1A3H2 monoclonal antibody in 50 mL/well of 0.05 M carbonate-bicarbonate buffer, pH 9.6, at 4 ºC overnight. The plates were washed thrice with PBS, pH 7.4, containing 0.05% Tween-20 (200 mL/well). Afterward, free binding sites were blocked by adding 150 mL/well of PBS containing 2% skimmed dry milk, previously inactivated by heating (100 ºC), and 0.01% Tween-20 for 1.5 h at 37 ºC, followed by washing as mentioned above. Standard or plasma (1:2000 diluted in PBS containing 1% skimmed milk and 0.01% Tween-20) was added (50 mL/well) in the plates and incubated for 1.5 h at 37 ºC. Then, the plates were washed and incubated with 0.5 mg/well of anti-LDL(-) 2C7D5F10 monoclonal antibody conjugated to biotin for one hour at 37 ºC. Afterward, 50 mL/well streptavidin-conjugated horseradish peroxidase (Invitrogen Corp., Carlsbad, CA, USA) was added and incubated for one hour at 37 ºC. The reactivity of peroxidase was measured on washed plates by incubating with 50 mL ortho-phenylenediamine (OPD) diluted in citrate phosphate buffer, pH 5.3, at 37 ºC for 15 min. The reaction was stopped by adding 2 M sulfuric acid, and the absorbance at 492 nm was measured by spectrophotometry using a microplate reader (Spectra Count Microplate Photometer, Packard Instruments Company, Downers Grove, IL, USA). A calibration curve (from 0.6 to 20 mg/mL) was built with human LDL(-) obtained by FPLC using an ionic column. Results were expressed as U/L, with units representing 1 g/L of oxidized apolipoprotein B equivalent.

### Red blood cell fatty acids analysis

The analysis of fatty acids (FA) from erythrocyte membranes was performed based on a previously described method.^18^After plasma separation (3000× *g* , 10 min, 4 °C), 300 µL of erythrocytes were washed with 5 mL of phosphate-buffered saline (PBS) solution (pH 7.4) four times. The precipitate was transferred to threaded tubes, to which 1.75 mL of methanol, 50 µL of an internal standard solution containing 1 mg tridecanoic acid (C13:0)/1 mL hexane, and 100 µL of acetyl chloride were added. Thereafter, the solution was vortexed and heated in a water bath at 90 °C for 1 hour. After that, 1.5 mL of hexane was added, and the solution was homogenized for 1 min. The samples were centrifuged at 1500× *g* , 4 °C for 2 min, and 800 µL of the supernatant was transferred to a different tube. This step was repeated with the addition of 750 µL of hexane. The tubes containing the collected supernatants were placed on a centrifugal concentrator at 40 °C for 20 min. Then, the FA methyl esters were dissolved in 150 µL of hexane and transferred to a glass insert in a vial, which subsequently went for gas chromatography analysis (Shimadzu CG-2010 equipped with a capillary column DB-FFAP, Agilent technologies).

The results were expressed as a percentage of the total FA. Analyses were conducted considering the fatty acids individually, as well as the total n-3, formed by the sum of α-linolenic acid (ALA; C18:3 n-3), eicosapentaenoic acid (EPA; C20:5 n-3), and docosahexaenoic acid (DHA; C22:6 n-3), and total n-6, formed by the sum of linoleic acid (LA; C18:2 n-6), γ-linolenic acid (GLA; C18:3 n-6), eicosadienoic acid (EDA; C20:2 n-6), di-homo-γ-linolenic acid (DGLA; C20:3 n-6), arachidonic acid (AA; C20:4 n-6), and di-homo-linoleic acid (DLA; C22:2 n-6).

### Statistical analyses

This study used convenience sampling based on secondary data from a previous randomized clinical trial (ReBEC: RBR-2vfhfv).

Continuous variables were expressed through mean ± standard deviation (SD) or median and interquartile range (IQR), according to data distribution, and categorical variables were expressed through absolute (n) or relative (%) frequency. The Kolmogorov-Smirnov test was performed on continuous variables to assess distribution. Data in sample characteristics were obtained through an independent t-test or Mann-Whitney U test for continuous variables according to data normality and a chi-square test for categorical parameters. Patients were divided into tertiles of RBC FA (total n-3, total n-6, and n-6/n-3 ratio), and then one-way ANOVA or Kruskal-Wallis tests were applied based upon the variable’s distribution, using Bonferroni as posthoc test.

Multiple linear regressions were conducted to associate FA with LDL(-), LDL size, and sdLDL-c, the latter being the dependent variable. All models were adjusted by age, sex, smoking status, and use of statins. All regression assumptions were fulfilled (i.e., no multicollinearity, homoscedasticity, normally distributed and independent errors, independence of the outcome variables, and linearity of the variables).

All tests were two-sided, with p < 0.05 considered significant, and performed on the SPSS software version 20.0.

## Results

Characteristics of participants are described in Table 1 and Supplementary Table 1 . Women reported a higher frequency of statin treatment and had levels of plasma total cholesterol, HDL-c, LDL-c, apoA-I, large, intermediate, and small HDL-c, LDL(-), and hs-CRP than men. In contrast, men had higher plasma triglycerides, sdLDL-c, and glucose. Compared to men, women had a higher intake of lipids (absolute values, but not relative intake), DGLA, and EPA and less energy (Supplementary Table 2 ). The analyzed RBC FA profile is described in Supplementary Table 3.

**Table 1:** – Sample characteristics

Variables	Total (n = 335)	

	Mean/median or n	SD/IQR or %
Age (years)	52.4	10.5
Physical activity (points)	7.2	1.4
Systolic blood pressure (mmHg)	133	18.3
Diastolic blood pressure (mmHg)	81.1	10
BMI (kg/m^2^)	30.9	5.7
Waist circumference (cm)	100.6	13.6
**Race**		
White	226	67.5
Non-white	109	12.5
**Smoking**		
Current smoker	64	19.1
Non-smoker	271	80.9
**Alcohol consumption**		
Yes	66	19.7
No	269	80.3
**Education**		
High school or less	191	57
College	144	43
**Non-communicable diseases**		
Diabetes Mellitus	66	19.7
Hypertension	189	56.4
Hypothyroidism	43	12.8
Dyslipidemia	186	55.5
**Medication**		
Statins	95	28.4
Antihypertensives	169	50.4
Antihyperglycemics	69	20.6
Fibrates	9	2.7
**Biochemical characteristics**		
Total cholesterol (mg/dL)	204.4	42
HDL-c (mg/dL)	36	30.0-43.0
LDL-c (mg/dL)	137.2	38.7
Triglycerides (mg/dL)	130	97.0-190.0
Non-HDL-c (mg/dL)	167.8	41.3
ApoA-I (mg/dL)	132.8	26
ApoB (mg/dL)	103.9	24.8
Large HDL-c (mg/dL)	10	7.0-14.0
Intermediate HDL-c (mg/dL)	18	15.0-21.0
Small HDL-c (mg/dL)	7.6	3
sdLDL-c (mg/dL)	3	1.0-9.0
lbLDL-c (mg/dL)	52.9	16.4
LDL size (Å)	270	266.0-272.0
LDL(-) (U/L)	5.3	1.8-17.9
Anti-LDL(-) (μg/mL)	8.1	5.00-11.5
Glucose (mg/dL)	98	91.0-108.0
Insulin (μUI/mL)	16.1	12.7-22.1
HbA1c (%)	5	4.7-5.3
Adiponectin (μg/mL)	8.3	4.7-13.0
Leptin (ng/mL)	34.6	11.0-65.4
hs-CRP (mg/L)	2.7	2.7-5.8

Data is shown in mean (standard deviation) or median (interquartile range) depending on the distribution for continuous data and absolute value (n) and frequency (%) for categorical data. ApoA-I: apolipoprotein A-I; ApoB: apolipoprotein B; BMI: body mass index; hs-CRP: high-sensitivity C-reactive protein; lbLDL-c: large buoyant LDL-cholesterol; LDL(-): electronegative LDL.

**Table 2:** – Associations between red blood cell membrane fatty acids and electronegative LDL, small dense LDL-cholesterol, and LDL particle size

Variables	LDL(-)				

	R^**2**^	β	SE	p-value	95% CI
C18:2 n-6 (LA)	0.034	0.551	0.779	0.480	-0.981 ₋ 2.083
C18:3 n-6 (GLA)	0.055	-6.071	3.643	0.097	-13.238 ₋ 1.096
C20:2 n-6 (EDA)	0.050	-7.125	7.121	0.318	-21.134 ₋ 6.885
C20:3 n-6 (DGLA)	0.065	10.724	4.253	**0.012**	2.356 ₋ 19.091
C20:4 n-6 (AA)	0.117	2.999	0.588	**< 0.001**	1.842 ₋ 4.157
C22:2 n-6 (DLA)	0.068	-12.869	4.760	**0.007**	-22.233 ₋ -3.506
Total n-6	0.075	1.163	0.367	**0.002**	0.440 ₋ 1.886
C18:3 n-3 (ALA)	0.054	-1.734	1.126	0.124	-3.949 ₋ 0.480
C20:5 n-3 (EPA)	0.061	-21.517	9.711	**0.027**	-40.621 ₋ -2.413
C22:6 n-3 (DHA)	0.048	-0.523	1.146	0.649	-2.777 ₋ 1.732
Total n-3	0.055	-1.443	0.850	0.090	-3.115 ₋ 0.229
n-6/n-3 ratio	0.072	4.064	1.364	**0.003**	1.381 ₋ 6.748
	**sdLDL-c**				

	**R^**2**^**	**β**	**SE**	**p-value**	**95% CI**
C18:2 n-6 (LA)	0.053	0.948	0.301	**0.002**	0.357 ₋ 1.540
C18:3 n-6 (GLA)	0.039	3.105	1.422	**0.030**	0.307 ₋ 5.903
C20:2 n-6 (EDA)	0.025	-0.263	2.792	0.925	-5.756 ₋ 5.230
C20:3 n-6 (DGLA)	0.049	4.824	1.654	**0.004**	1.569 ₋ 8.078
C20:4 n-6 (AA)	0.037	0.491	0.237	**0.039**	0.025 ₋ 0.958
C22:2 n-6 (DLA)	0.030	-2.405	1.835	0.191	-6.015 ₋ 1.205
Total n-6	0.053	0.453	0.144	**0.002**	0.170 ₋ 0.736
C18:3 n-3 (ALA)	0.052	-1.340	0.437	**0.002**	-2.199 ₋ -0.481
C20:5 n-3 (EPA)	0.031	-5.492	3.827	0.152	-13.020 ₋ 2.036
C22:6 n-3 (DHA)	0.026	-0.283	0.447	0.527	-1.163 ₋ 0.597
Total n-3	0.049	-0.958	0.329	**0.004**	-1.606 ₋ -0.311
n-6/n-3 ratio	0.062	1.905	0.530	**< 0.001**	0.863 ₋ 2.947
	**LDL particle size**				

	**R^**2**^**	**β**	**SE**	**p-value**	**95% CI**
C18:2 n-6 (LA)	0.079	-0.504	0.160	**0.002**	-0.818 ₋ -0.190
C18:3 n-6 (GLA)	0.060	-1.313	0.758	0.084	-2.805 ₋ 0.179
C20:2 n-6 (EDA)	0.051	0.069	1.485	0.963	-2.851 ₋ 2.990
C20:3 n-6 (DGLA)	0.065	-1.955	0.884	**0.028**	-3.695 ₋ -0.215
C20:4 n-6 (AA)	0.061	-0.232	0.126	0.067	-0.480 ₋ 0.017
C22:2 n-6 (DLA)	0.055	1.007	0.977	0.303	-0.914 ₋ 2.928
Total n-6	0.075	-0.224	0.077	**0.004**	-0.374 ₋ -0.073
C18:3 n-3 (ALA)	0.077	0.699	0.232	**0.003**	0.242 ₋ 1.156
C20:5 n-3 (EPA)	0.060	3.465	2.032	0.089	-0.533 ₋ 7.462
C22:6 n-3 (DHA)	0.053	0.157	0.238	0.511	-0.311 ₋ 0.624
Total n-3	0.075	0.510	0.175	**0.004**	0.165 ₋ 0.854
n-6/n-3 ratio	0,089	-1,032	0,281	**< 0,001**	-1,585 ₋ -0,478

Multiple linear regressions models adjusted by age, sex, smoking status, and use of statins. AA: arachidonic acid; ALA: α-linolenic acid; DGLA: dihomo-γ-linoleic acid; DHA: docosahexaenoic acid; DLA: di-homo-linoleic acid; EDA: eicosadienoic acid; EPA: eicosapentaenoic acid; GLA: γ-linolenic acid.

Individuals of the third tertile of total n-3 had significantly lower plasma apoB-associated lipoproteins cholesterol, triglycerides, and apoB levels. Also, better qualitative aspects of LDL were observed in this group, such as lower sdLDL-c (p = 0.001) and larger LDL particle size (p = 0.003) (Supplementary Table 4).

Associations found with RBC total n-6 PUFA were oppositely compared to n-3 PUFA (Supplementary Table 5). It was found that individuals within the third tertile had higher plasma total cholesterol, LDL-c, non-HDL-c, apoB, and apoA-I. As regards LDL particle quality, the third tertile had higher plasma levels of sdLDL-c (p = 0.004), lbLDL-c (p = 0.037), and LDL(-) (p = 0.002). Additionally, the mean size of LDL particles was smaller in this tertile (p = 0.026). Similar associations were found with the third tertile of the RBC n-6/n-3 ratio (Supplementary Table 6).

Associations of n-3 and n-6 PUFA with LDL particle quality are shown in Table 2 . EPA was inversely associated with LDL(-). ALA was significantly associated with lower sdLDL-c and larger LDL particle size, influencing the association of total n-3 with these parameters. Regarding n-6 PUFA, total n-6 had positive associations with LDL(-) and sdLDL-c, while it had an inverse association with LDL particle size. Among the associations with LDL(-), DGLA and AA showed significant positive associations, while DLA showed an inverse association. Among sdLDL-c associations, LA, GLA, DGLA, and AA showed positive associations. Furthermore, LDL particle size was inversely associated only with LA and DGLA among n-6 PUFA. Finally, the RBC n-6/n-3 PUFA ratio was positively associated with LDL(-) and sdLDL-c and inversely associated with LDL particle size.

## Discussion

This study showed that RBC n-6 and n-3 PUFA had opposing influences on cardiometabolic health markers and LDL particle characteristics.

Our results showed that individuals with higher levels of RBC n-6 PUFA and n-6/n-3 ratio and lower levels of n-3 PUFA had higher plasma cholesterol (total, LDL-c, and non-HDL-c), apoB, apoA-I, and triglycerides. These results are consistent with studies showing that a high n-6/n-3 ratio was associated with increased lipogenesis and blood lipids.^19 - 24^

Clinical studies of n-6 PUFA biomarkers and CVD in the literature show inconclusive results. In a previous study, an RBC FA pattern containing more n-6 PUFA was an independent predictor of higher cardiovascular risk classification associated with established risk factors.^18^Conversely, tissue or blood n-6 PUFA were not associated with cardiovascular risk.^25 , 26^Moreover, studies have found that high n-6 PUFA, especially LA, were protective factors against acute coronary syndrome.^27 - 29^Authors of one of these studies stated that n-6 PUFA consumption should be encouraged despite the low consumption found in one study.^29^Furthermore, it is important to note that the risk for CV outcomes had a U-shaped association with AA,^28^and LA was not significantly associated with myocardial infarction risk in one of these studies,^29^making it difficult to conclude the real effect of n-6 PUFA on CVD.

Evidence suggests that n-6 PUFA reduces plasma cholesterol, particularly when SFA is replaced, which is favorable for CVD risk reduction,^30 - 32^but the effects on outcomes remain inconclusive.^31^A meta-analysis of randomized controlled trials (RCTs) showed protective effects of LA on CVD using vegetable oils, which are also a source of ALA.^33^Interestingly, one RCT reported an increased risk for CVD and all-cause mortality after n-6 PUFA supplementation.^33^Furthermore, results of the Lyon Diet Heart Study showed that the reduction of n-6 PUFA to less than 5% of total energy intake, below the US recommendations of 5-10%, decreased total and cardiovascular mortality, suggesting that there is a risk of the consumption associated with n-6 PUFA.^34 , 35^

Concerning n-3 PUFA, the recent clinical evidence for CVD is more consistent. Observational studies with RBC n-3 PUFA^18 , 36 - 40^and randomized clinical trials^41 - 43^showed that n-3 PUFA reduce CVD risk. Our results corroborate with the cardioprotective effect of n-3 PUFA but showed a strong influence of ALA due to the low EPA and DHA intake (Supplementary Table 1 ). Our study’s results align with a systematic review and dose-response meta-analysis, which showed that a higher intake of ALA was significantly associated with 10%, 8%, and 11% of mortality from all causes, CVD, and CAD, respectively.^44^

Traditional cardiovascular risk factors do not reflect the full complexity of the atherosclerotic disease, so much so that emerging cardiovascular risk factors have gained emphasis in the literature,^18 , 45 - 48^and the effects of PUFA on CVD are beyond these factors.^18^Among the various non-traditional risk factors, it has been shown that sdLDL-c is a better predictor of CAD risk than LDL-c.^1^The current study found that RBC n-6 PUFA and n-6/n-3 ratio were positively associated with smaller average LDL particle size and higher levels of sdLDL-c. Similar associations were observed with LA, GLA, DGLA, and AA individually. In contrast to our findings, a cross-sectional study showed that circulating n-6 PUFA was modestly associated with LDL size.^49^

Conversely, n-3 PUFA were associated with larger LDL particle size and lower levels of sdLDL-c. Such associations corroborate previous trials’ results that n-3 PUFA decreased sdLDL-c and increased LDL particle size.^50 - 53^These associations may be linked to TG. Higher TG increases apoC-III, which hinders clearance of apoE-containing lipoproteins, resulting in increased circulating sdLDL levels,^1^and n-3 PUFA reduce apoC-III levels in VLDL, decreasing sdLDL levels.^54^

We also found that RBC n-6/n-3 ratio, total n-6 PUFA, DGLA, and AA were positively associated with LDL(-), whereas EPA and DLA presented an inverse association. LDL(-), closely related to oxLDL and sdLDL, has atherogenic properties, and increased levels of this particle are found in oxidative stress conditions and non-communicable diseases.^1 , 2^High n-6/n-3 tissue levels are associated with increased lipid peroxidation,^19 - 21^and n-6 PUFA in LDL particles, primarily LA and AA, are most readily oxidized, resulting in the increased formation of modified LDL particles.^12^Another study showed that lowering dietary n-6 PUFA reduces its metabolites generated from peroxidation.^55^Interestingly, we found that EPA was a protective factor. This may be due to the anti-inflammatory and antioxidant mechanism of EPA and its metabolites, which decrease the modification of LDL particles.^56^

Among limitations, we cannot infer causality because of the study’s cross-sectional nature. In addition, RBC PUFA in our study differs from previous results.^48 , 55 , 57^This may be due to a lack of standardization of circulating FA analysis. RBC FA are expressed in percentages of total FA, meaning that the FA levels depend on FA analyzed by the method. Another factor that may explain this divergence is that oxidative stress decreases the amount of PUFA incorporated into the tissues.^58^Finally, our study does not have a representative sample, so RBC FA and their association with cardiovascular risk factors may differ from other populations.

Our findings add to the knowledge about fatty acids and cardiovascular risk, especially regarding n-6 PUFA. The literature proposes that higher n-6 PUFA intake may benefit individuals concerning cardiovascular risk.^7 - 10^However, the studies show very inconsistent results since different biomarkers (e.g., plasma, RBC, adipose tissue, dietary) are evaluated, and the diet of the individuals may vary greatly among the regions studied (e.g., Western countries vs. Asian countries). With the association of RBC n-6 PUFA and the n-6/n-3 ratio with LDL(-) that was demonstrated, our study corroborates with LDL oxidation experiments that showed that the oxidized FA are from the n-6 PUFA family,^11 - 14^suggesting that excess n-6 PUFA consumption may cause adverse effects. This calls attention to the fact that the benefits found from LA and other n-6 PUFA in the literature probably depend on the nutritional status of n-3 PUFA since we showed favorable metabolic associations with the latter.

This study finally shows that upcoming studies of the nutritional status of PUFA should consider both families of fatty acids since analysis alone may ignore confounding factors. Further studies are required to evaluate the association of RBC PUFA with cardiovascular risk factors and outcomes, as well as emerging risk factors such as LDL subfractions.

## Conclusion

Our study showed RBC n-6 PUFA were associated with worse cardiometabolic risk factors and higher levels of sdLDL-c and LDL(-), suggesting that high dietary n-6 PUFA increases LDL susceptibility to oxidative modifications. In turn, RBC n-3 PUFA, especially ALA and EPA, showed cardioprotective associations, with lower levels of sdLDL-c and LDL(-) and larger LDL particle size.

Finally, n-6 PUFA intake (both LA or AA) should be decreased at the same time as the n-3 PUFA should be stimulated since a higher RBC n-6/n-3 ratio and n-6 PUFA have been associated with worse LDL particle quality and cardiometabolic risk factors.

## Supplemental Materials

For supplementary tables, please click here .
